# Prevalence and Antibiotic‐Resistance Profile of MRSA and MSSA on High‐Touch Hospital Surfaces in the Buea Health District, Cameroon

**DOI:** 10.1155/bmri/1825277

**Published:** 2025-11-04

**Authors:** Dany Mayeul Nseufeu Yomi, Seraphine Nkie Esemu, Jerome Achah Kfusi, Lucy Mande Ndip, Raymond Babila Nyasa

**Affiliations:** ^1^ Department of Microbiology and Parasitology, University of Buea, Buea, Cameroon, ubuea.cm; ^2^ Laboratory for Emerging Infectious Diseases, University of Buea, Buea, South West Region, Cameroon, ubuea.cm

**Keywords:** antibiotic resistance, Buea Health District, high-touch surfaces, MRSA, *Staphylococcus aureus*

## Abstract

**Background:**

*Staphylococcus aureus* is a major cause of hospital‐acquired infections, with methicillin‐resistant strains posing significant treatment challenges. High‐touch hospital surfaces can serve as reservoirs for *S. aureus*, facilitating transmission among patients and healthcare workers. This study investigated the prevalence and antibiotic‐resistance profile of methicillin‐resistant (MRSA) and methicillin‐susceptible *Staphylococcus aureus* (MSSA) on high‐touch hospital surfaces in the Buea Health District, Cameroon.

**Methods:**

A cross‐sectional study was conducted in six randomly selected hospitals. Swab samples were collected from bedrails, tabletops, door handles, chairs, and light switches. *S. aureus* isolates were identified by mannitol fermentation, Gram staining, and catalase testing, and confirmed by detection of the *nuc* gene. Methicillin resistance was determined by amplification of the *mecA* gene. Antibiotic susceptibility was assessed using the Kirby–Bauer disk diffusion method against 10 commonly used antibiotics.

**Results:**

Of 327 samples, 15 (4.6%) were confirmed as *S. aureus*, comprising 1 (0.3%) MRSA and 14 (4.3%) MSSA. Contamination was highest on bedrails, followed by tabletops and door handles. The isolates were highly susceptible to gentamicin (100%), ceftriaxone (86.7%), cefoxitin (86.7%), and doxycycline (86.7%), but showed complete resistance to ampicillin (100%). Four isolates, including the MRSA strain, were multidrug resistant.

**Conclusion:**

High‐touch hospital surfaces in the Buea Health District are contaminated with MRSA and MSSA. These findings highlight the need for enhanced infection prevention and control measures to reduce transmission in hospital settings.

## 1. Introduction

*Staphylococcus aureus* is a major cause of hospital‐acquired infections, ranging from mild skin and soft tissue infections to severe, life‐threatening conditions such as pneumonia, bacteremia, and endocarditis [1]. Although part of the normal human flora, *S. aureus* becomes pathogenic when skin or mucosal barriers are breached, particularly in vulnerable patients with weakened immunity or invasive medical devices [2, 3]. Some strains, notably methicillin‐resistant *S. aureus* (MRSA), have developed resistance to antibiotics, making infections increasingly difficult to treat [4].

MRSA poses a serious public health threat due to its resistance to *β*‐lactam antibiotics. This resistance is mediated by the *mecA* gene, located on the staphylococcal cassette chromosome mec (SCCmec), which encodes an altered penicillin‐binding protein (PBP2a) with reduced affinity for *β*‐lactams antibiotics [5]. In hospital settings, MRSA infections result in prolonged hospital stays, increased healthcare costs, and higher morbidity and mortality [3, 6]. These challenges are particularly concerning in resource‐limited settings, where inadequate infection control measures and limited diagnostic capacity increase the risk of MRSA transmission [7].

High‐touch hospital surfaces, such as bedrails, door handles, and tabletops, are frequently contaminated with *S. aureus*, including MRSA [6, 8, 9]. Patients colonized or infected with *S. aureus* can shed the bacteria onto these surfaces, where they may persist for extended periods. Contaminated surfaces thus serve as reservoirs, facilitating transmission to other patients and healthcare workers [5, 10]. A meta‐analysis reported that approximately 15% of hospital surfaces were contaminated with *S. aureus*, with around 5% harboring MRSA [11].

Although MRSA is among the most widely reported antibiotic‐resistant organisms globally [12], few studies have investigated its presence on hospital surfaces in Cameroon. Most existing research has focused on clinical samples [13–15], leaving a gap in understanding the role of environmental contamination in MRSA transmission. This study investigated the prevalence and antibiotic‐resistance profile of MRSA and methicillin‐susceptible *S. aureus* (MSSA) on high‐touch hospital surfaces in the Buea Health District, Cameroon, to provide evidence to guide infection prevention and control efforts in the study area.

## 2. Materials and Methods

### 2.1. Study Area

This study was conducted in the Buea Health District. Buea, the administrative capital of the South West Region of Cameroon, has an estimated population of 300,000 [16]. The Buea Health District encompasses both urban and rural communities and is divided into seven health areas: Bokwango, Bova, Buea Road, Buea Town, Molyko, Muea, and Tole. It comprises 27 health facilities of varying capacities [17].

### 2.2. Study Design

This was a cross‐sectional, hospital‐based study carried out from April to August 2023 to determine the prevalence and antibiotic‐resistance profile of MRSA and MSSA on high‐touch hospital surfaces. Samples were collected from six randomly selected hospitals in the Buea Health District: Solidarity Clinic (SC), Police Medical Center (PMC), Buea Regional Hospital (BRH), Molyko Integrated Health Center (MIHC), Centre Médical d’Arrondissement Muea (CMA), and Seventh‐day Adventist Health Center (SAHC). Laboratory procedures were performed at the Laboratory for Emerging Infectious Diseases, University of Buea.

### 2.3. Ethical Considerations

Ethical approval was obtained from the Institutional Review Board of the Faculty of Health Sciences, University of Buea (Ref: 2023/2020‐03/UB/SG/IRB/FHS). Administrative authorization was granted by the South West Regional Delegation of Public Health (Ref: R11/MINSANTE/SWR/RDPH/PS/715/620), and permission was obtained from all participating hospitals.

### 2.4. Determination of Sample Size

The minimum sample size was calculated using Fisher’s formula [18].

n=Z2 P1−Pd2

where *n* = minimum sample size, *Z* = 1.96 (standard normal deviate at 95% confidence), *P* = estimated prevalence, and *d* = degree of precision (0.05). A study in Tanzania that reported 19.5% MRSA prevalence on high‐touch surfaces was used as the estimate [6].

n=1.962×0.1950.8050.052=241

The minimum required sample size was 241; however, 327 samples were collected.

### 2.5. Sample Collection

Samples were collected from high‐touch hospital surfaces (bedrails, tabletops, door handles, light switches, and chairs) in patient rooms, few hours after routine daily cleaning and disinfection. Sterile cotton swabs pre‐moistened with sterile normal saline (0.85% NaCl, w/v) were used to swab an area of approximately 20 cm^2^ per surface. Swabs were placed in sterile tubes, labeled with the date, hospital unit, and surface type, transported on ice, and processed in the laboratory within 1 h of collection.

### 2.6. Isolation of *Staphylococcus aureus*

Swabs were enriched in 5 mL of nutrient broth and incubated at 37°C for 24 h. After incubation, 100 *μ*L aliquots were spread onto mannitol salt agar and incubated again at 37°C for 24 h. Colonies that fermented mannitol, indicated by yellow coloration of the medium, were Gram‐stained. Gram‐positive cocci arranged in clusters were subcultured on nutrient agar and incubated at 37°C for 24 h. Pure colonies were then screened for catalase production; catalase‐positive isolates were considered presumptive *S. aureus*. The presumptive isolates were preserved by inoculating a loopful of culture into sterile Eppendorf tubes containing 1 mL of nutrient broth. The suspensions were vortexed, incubated at 37°C for 24 h, and centrifuged at 10,000 rpm for 5 min. Eight hundred microliters of the supernatant were discarded and replaced with 800 *μ*L of 50% glycerol. The mixtures were vortexed thoroughly and stored at – 20°C for further analysis.

### 2.7. DNA Extraction

Genomic DNA was extracted using the boiling method adapted from Ndedy et al. [15]. Presumptive isolates were revived on nutrient agar and incubated at 37°C for 24 h. Five colonies from each isolate were suspended in 200 *μ*L of nuclease‐free water and vortexed for 20 s. The suspensions were boiled in a water bath at 100°C for 15 min, chilled on ice for 15 min, and thawed at 37°C for another 15 min. This freeze–thaw cycle was repeated once. The lysates were centrifuged at 14,000 rpm for 5 min, and 150 *μ*L of the supernatant was transferred into sterile Eppendorf tubes. DNA presence in the supernatant was confirmed by electrophoresis on a 1.5% agarose gel stained with 0.5 *μ*g/mL ethidium bromide and visualized under ultraviolet light.

### 2.8. Molecular Identification of *Staphylococcus aureus*

Confirmation of *S. aureus* was performed by PCR amplification of the *nuc* gene, as previously described [19]. Each 25 *μ*L PCR reaction contained 5 *μ*L of DNA template, 12.5 *μ*L of master mix, 6.5 *μ*L of nuclease‐free water, and 0.5 *μ*L each of forward (5⁣^′^‐AGCCAAGCCTTGACGAACTAAAGC‐3⁣^′^) and reverse (5⁣^′^‐GCGATTGATGGTGATACGGTT‐3⁣^′^) primers. A negative control containing nuclease‐free water instead of DNA template was included in each run. Amplification of a 280 bp fragment of the *nuc* gene was carried out under the following cycling conditions: 1 cycle of initial denaturation at 95°C for 5 min; 40 cycles of denaturation at 94°C for 1 min, annealing at 55°C for 1 min, and extension at 72°C for 1 min, followed by a final extension at 72°C for 5 min. PCR products were electrophoresed on a 1.5% agarose gel stained with 0.5 *μ*g/mL ethidium bromide, visualized under ultraviolet light and photographed using Gel Documentation‐XR (BIORAD, Hercules, California).

### 2.9. Identification of MRSA

Methicillin resistance was determined in *S. aureus* isolates by PCR amplification of the *mecA* gene, as previously described [19]. The PCR setup and conditions were the same as described for *nuc* gene amplification, with the following differences: *mecA* primers (forward: 5⁣^′^‐AAAATCGATAAAGGTTGGC‐3⁣^′^; reverse: 5⁣^′^‐AGTTCGCAGTTACCGGATTTGC‐3⁣^′^), 35 cycles, and an annealing temperature of 50°C. Amplification of a 533 bp fragment confirmed the presence of *mecA*.

### 2.10. Antibiotic Susceptibility Testing

The antibiotic‐resistance profile of MRSA and MSSA isolates was determined using the Kirby–Bauer disk diffusion method, following Clinical and Laboratory Standards Institute (CLSI) guidelines [20]. Ten antibiotics were tested: ampicillin (10 *μ*g), cefoxitin (30 *μ*g), ceftriaxone (30 *μ*g), chloramphenicol (30 *μ*g), ciprofloxacin (5 *μ*g), clindamycin (2 *μ*g), doxycycline (30 *μ*g), erythromycin (15 *μ*g), gentamicin (10 *μ*g), and ofloxacin (5 *μ*g). Bacterial suspensions were prepared in sterile normal saline (0.85% NaCl, w/v) and adjusted to the 0.5 McFarland standard (approximately 1.5 × 10^8^ CFU/mL). One hundred microliters of each suspension were spread evenly onto Mueller–Hinton agar plates, and antibiotic disks were applied using sterile forceps with at least 24 mm spacing. The plates were incubated at 35°C for 24 hours, and inhibition zones were measured and interpreted as susceptible, intermediate, or resistant according to CLSI breakpoints [20].

### 2.11. Data Analysis

Data was analyzed using SPSS version 23.0. Descriptive statistics were used to determine the prevalence of MRSA and MSSA, as well as their antibiotic‐resistance profile. The chi‐square test was used to compare contamination rates across surface types, with *p* value < 0.05 considered statistically significant.

## 3. Results

### 3.1. Distribution of Samples

A total of 327 samples were collected from five high‐touch surfaces across six hospitals in the Buea Health District. Of these, 66 (20.2%) were collected from SC, 17 (5.2%) from PMC, 125 (38.2%) from BRH, 31 (9.5%) from MIHC, 42 (12.8%) from CMA, and 46 (14.1%) from SAHC. By surface type, 105 (32.1%) were collected from bedrails, 70 (21.4%) from tabletops, 51 (15.6%) from door handles, 46 (14.1%) from light switches, and 55 (16.8%) from chairs.

### 3.2. Isolation and Identification of *Staphylococcus aureus* and MRSA

Of the 327 samples analyzed, 124 yielded colonies with phenotypic characteristics typical of *S. aureus* on mannitol salt agar. These colonies fermented mannitol, were Gram‐positive cocci in clusters, and catalase‐positive (Figure 1). PCR amplification of the *nuc* gene (280 bp) confirmed 15 isolates as *S. aureus*. Of these, one isolate was identified as MRSA through amplification of the *mecA* gene (533 bp) (Figure 2).

Figure 1Identification of *S. aureus*. (a) Colony growth on mannitol salt agar. (b) Gram‐positive cocci in clusters. (c) Catalase test.

**Figure:**
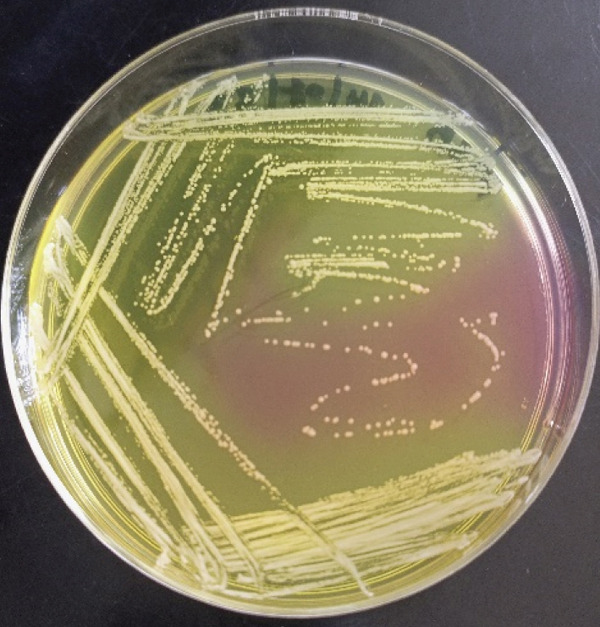
(a)

**Figure:**
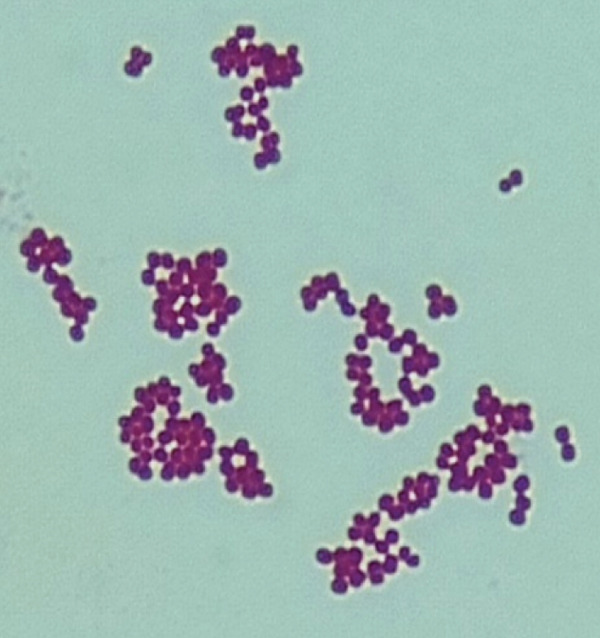
(b)

**Figure:**
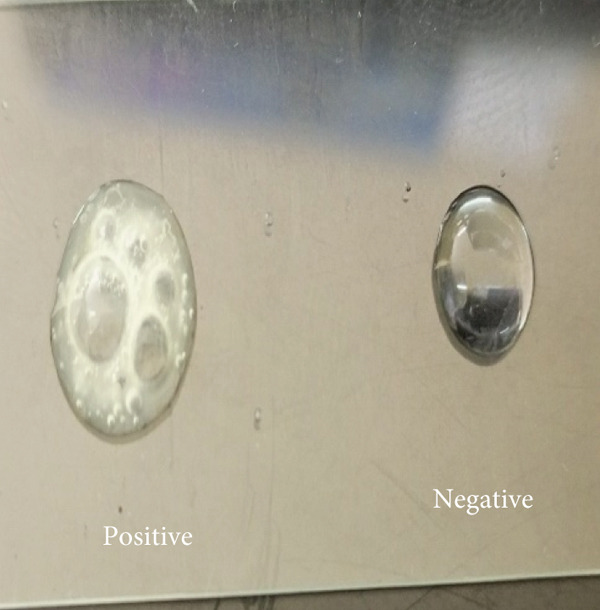
(c)

Figure 2Visualization of amplified PCR products on 1.5% agarose gel after electrophoretic separation at 90 V for 1 h. (a) *nuc* gene PCR products: 100 bp molecular weight marker (lane 1), negative control (lane 2), positive samples (lanes 3–8). (b) *mecA* gene PCR products: 100 bp molecular weight marker (lane 1), negative control (lane 2), positive sample (lane 4), and negative samples (lanes 3 and 5).

**Figure:**
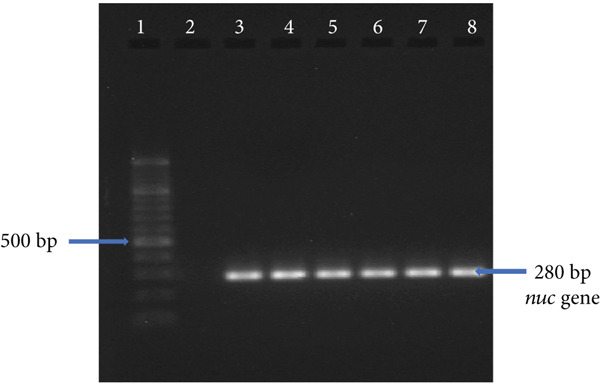
(a)

**Figure:**
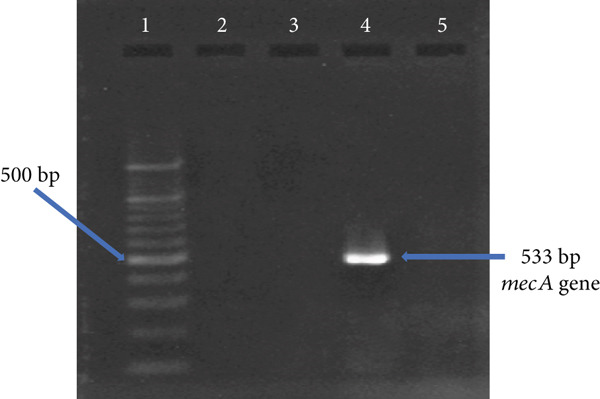
(b)

### 3.3. Prevalence of MRSA and MSSA

The overall prevalence of *S. aureus* was 4.6% (15/327), comprising 0.3% (1/327) MRSA and 4.3% (14/327) MSSA. Of the 15 S*. aureus* isolates identified, 7 were recovered from BRH, 4 from CMA, 3 from SC, and 1 from MIHC. No isolates were detected in samples from SAHC or PMC. By surface types, 11 isolates were found on bedrails, 3 on tabletops, and 1 on a door handle, while none were detected on chairs or light switches (Table 1). A chi‐square test revealed a statistically significant difference in contamination across surface types (*X* ^2^ = 13.99; *p* = 0.007) with higher contamination observed on bedrails and tabletops.

**Table 1:** Distribution of *Staphylococcus aureus* isolates with respect to surface types and hospitals.

	**Number of samples**	**Number of *S. aureus* isolates**	**Number of MSSA isolates**	**Number of MRSA isolates**
Surface types				
Bedrails	105	11	10	1
Tabletops	70	3	3	0
Door handles	51	1	1	0
Light switches	46	0	0	0
Chairs	55	0	0	0
Total	327	15	14	1
Hospitals				
BRH	125	7	6	1
CMA	42	4	4	0
SC	66	3	3	0
MIHC	31	1	1	0
SAHC	46	0	0	0
PMC	55	0	0	0
Total	327	15	14	1

### 3.4. Antibiotic‐Resistance Profiles of the Isolates

**Figure 3:**
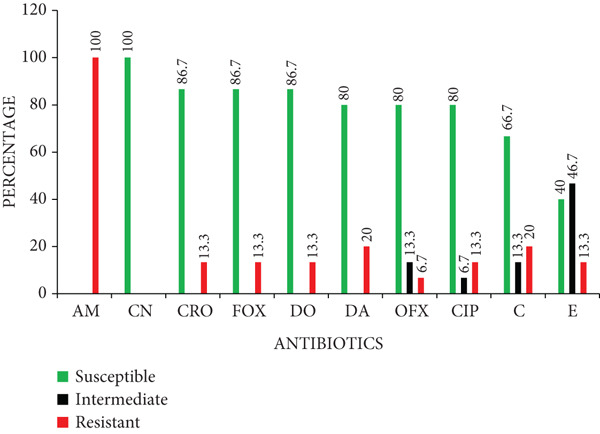
Antibiotic‐resistance profile of *Staphylococcus aureus* isolates. Key: AM, ampicillin; CN, gentamicin; CRO, ceftriaxone; FOX, cefoxitin; DO, doxycycline; DA, clindamycin; OFX, ofloxacin; CIP, ciprofloxacin; C, chloramphenicol; E, erythromycin.

Antibiotic susceptibility testing showed that all 15 S*. aureus* isolates were susceptible to gentamicin (100%). High susceptibility was also observed to ceftriaxone (86.7%), cefoxitin (86.7%), doxycycline (86.7%), clindamycin (80.0%), ofloxacin (80.0%), and ciprofloxacin (80.0%). Moderate susceptibility was recorded for chloramphenicol (66.7%), whereas erythromycin showed low susceptibility (40.0%). On the contrary, all isolates were resistant to ampicillin (100%). A few isolates showed intermediate resistance to erythromycin, ofloxacin, chloramphenicol, and ciprofloxacin (Figure 3).

### 3.5. Resistance Patterns and Multidrug Resistance

Among the 15 S*. aureus* isolates, 10 were resistant to a single antibiotic and 1 isolate was resistant to two antibiotics. Four isolates, including the MRSA strain, were classified as multidrug resistant, defined as resistance to three or more antibiotic classes (Table 2).

**Table 2:** Resistance patterns and antibiotypes of *S. aureus* isolates.

**Antibiotypes**	**No. of isolates**	**Methicillin status**	**Resistance category**	**Sample source**
AM	10	MSSA	Resistant to one antibiotic	Bedrails, tabletops, door handles
AM‐C	1	MSSA	Resistant to two antibiotics	Bedrail
AM‐C‐DA	1	MSSA	Multidrug‐resistant	Bedrail
AM‐C‐CIP	1	MSSA	Multidrug‐resistant	Bedrail
AM‐DA‐CRO‐E‐DO‐FOX	1	MSSA	Multidrug‐resistant	Bedrail
AM‐CIP‐DA‐CRO‐E‐DO‐OFX‐FOX	1	MRSA	Multidrug‐resistant	Bedrail
**Total**	**15**			

Abbreviations: AM, ampicillin; C, chloramphenicol; CIP, ciprofloxacin; CRO, ceftriaxone; DA, clindamycin; DO, doxycycline; E, erythromycin; FOX, cefoxitin; OFX, ofloxacin.

## 4. Discussion

*Staphylococcus aureus* remains a major cause of hospital‐acquired infections worldwide. Its ability to persist on hospital surfaces and its potential for multidrug resistance make it a significant threat to patient safety and infection control. This study was carried out to determine the prevalence and antibiotic‐resistance profile of MRSA and MSSA on high‐touch hospital surfaces in the Buea Health District, Cameroon. The prevalence of MRSA and MSSA observed in this study was lower than that reported in other African settings, where contamination ranges from 10 to 25% for MSSA and up to 20% for MRSA [6, 8, 21, 22]. These differences in prevalence may reflect variations in study design, sample size, types of surfaces sampled, laboratory methods, and infection control practices.

Contamination levels varied significantly across surface types. Bedrails exhibited the highest contamination rates, followed by tabletops and door handles, whereas chairs and light switches had no detectable contamination. The predominance of bedrail contamination aligns with findings from Tanzania, South Africa, and Nepal, where bedrails were also reported as the most contaminated surfaces [6, 8, 23], likely due to their high contact frequency because they are in close proximity to patients. This highlights the need for targeted disinfection of surfaces near patients.

The majority of *S. aureus* isolates were recovered from hospitals with high patient throughput, particularly Buea Regional Hospital, the district’s largest referral center, followed by Centre Médical d’Arrondissement Muea and Solidarity Clinic. In contrast, facilities with lower patient volumes showed minimal contamination. These findings suggest that hospitals with higher patient loads may be at greater risk of environmental contamination, highlighting the need to strengthen infection prevention and control measures in such settings.

Antibiotic resistance remains a serious global health concern, and the spread of resistant staphylococci, particularly MRSA, poses a major challenge to infection management. In this study, MSSA isolates were highly susceptible to gentamicin, ceftriaxone, cefoxitin, doxycycline, clindamycin, ofloxacin, and ciprofloxacin. This suggests that these antibiotics are not commonly overused in the Buea Health District and may therefore be recommended for the treatment of MSSA infections. On the Contrary, all isolates were resistant to ampicillin, reflecting widespread *β*‐lactamase production in *S. aureus*, an enzyme that hydrolyzes the *β*‐lactam ring in penicillins [24]. The single MRSA isolate identified was resistant to eight of 10 antibiotics tested, retaining susceptibility only to gentamicin and chloramphenicol. This pattern is consistent with other studies highlighting limited treatment options for MRSA infections [15, 19, 25]. Moreover, the detection of multidrug‐resistant MSSA strains underscores the growing threat of antimicrobial resistance in the region and calls for strengthened antibiotic stewardship programs.

### 4.1. Limitations of the Study

The inclusion of only six hospitals may limit the generalizability of these findings to the entire Buea Health District. Sampling was restricted to five high‐touch surface types, potentially overlooking other *S. aureus* reservoirs. Molecular analyses were restricted to the *nuc* and *mecA* genes, and other resistance genes, such as *mecC* were not assessed, which may underestimate MRSA prevalence. In addition, the cross‐sectional design of this study provides only a snapshot of surface contamination at a single point in time rather than capturing longitudinal trends.

The study was also limited by variability in the availability of hospital units. While some hospitals had all five units (medical, surgical, pediatric, maternity, and intensive care), others lacked certain units, and in some cases, medical and surgical units were combined. Consequently, we could not determine which specific units were more likely to harbor surfaces contaminated with *S. aureus.*

## 5. Conclusion

High‐touch hospital surfaces in the Buea Health District are contaminated with MRSA and MSSA. While most isolates remain susceptible to several antibiotics, complete resistance to ampicillin and the detection of multidrug‐resistant strains highlight the need for enhanced infection prevention and control measures, as well as continuous antibiotic resistance surveillance to reduce the spread of these pathogens in hospital settings. Future studies should investigate *S. aureus* contamination across a wider range of hospital surfaces and facilities, include screening for additional resistance genes, and adopt longitudinal sampling to better understand surface‐mediated transmission in Cameroonian hospitals.

## Ethics Statement

Ethical approval to carry out this study was obtained from the Institutional Review Board of the Faculty of Health Sciences, University of Buea (Ref: 2023/2020‐03/UB/SG/IRB/FHS). Administrative authorization was obtained from the South West Regional Delegation of Public Health (Ref No. R11/MINSANTE/SWR/RDPH/PS/715/620). Permission was obtained from all participating hospitals.

## Disclosure

All authors read and approved the final manuscript.

## Conflicts of Interest

The authors declare no conflicts of interest.

## Author Contributions

D.M.N.Y. and S.N.E. conceived and designed the study; S.N.E. and R.B.N. supervised the study; D.M.N.Y. collected samples; D.M.N.Y. and J.A.K. performed the experiment; D.M.N.Y., R.B.N., S.N.E., and L.M.N. performed statistical/data analysis; D.M.N.Y. and R.B.N. drafted the manuscript.

## Funding

No funding was received for this manuscript.

## Data Availability

The data that supports the findings of this study are available from the corresponding author upon reasonable request.
